# Single-cell RNA-sequencing reveals transcriptional dynamics of estrogen-induced dysplasia in the ovarian surface epithelium

**DOI:** 10.1371/journal.pgen.1007788

**Published:** 2018-11-12

**Authors:** Nhung H. Vuong, David P. Cook, Laura A. Forrest, Lauren E. Carter, Pascale Robineau-Charette, Joshua M. Kofsky, Kendra M. Hodgkinson, Barbara C. Vanderhyden

**Affiliations:** 1 Department of Cellular and Molecular Medicine, University of Ottawa, Ottawa, Canada; 2 Cancer Therapeutics Program, Ottawa Hospital Research Institute, Ottawa, Canada; Stanford University School of Medicine, UNITED STATES

## Abstract

Estrogen therapy increases the risk of ovarian cancer and exogenous estradiol accelerates the onset of ovarian cancer in mouse models. Both *in vivo* and *in vitro*, ovarian surface epithelial (OSE) cells exposed to estradiol develop a subpopulation that loses cell polarity, contact inhibition, and forms multi-layered foci of dysplastic cells with increased susceptibility to transformation. Here, we use single-cell RNA-sequencing to characterize this dysplastic subpopulation and identify the transcriptional dynamics involved in its emergence. Estradiol-treated cells were characterized by up-regulation of genes associated with proliferation, metabolism, and survival pathways. Pseudotemporal ordering revealed that OSE cells occupy a largely linear phenotypic spectrum that, in estradiol-treated cells, diverges towards cell state consistent with the dysplastic population. This divergence is characterized by the activation of various cancer-associated pathways including an increase in *Greb1* which was validated in fallopian tube epithelium and human ovarian cancers. Taken together, this work reveals possible mechanisms by which estradiol increases epithelial cell susceptibility to tumour initiation.

## Author summary

Women who take estrogen replacement therapy are at higher risk of developing ovarian cancer. When ovarian epithelial cells are exposed to estrogen, there is a heterogeneous cellular response, with some cells appearing unaffected, while others become disorganized and grow at accelerated rates consistent with pre-cancerous cells. This heterogeneity confounds traditional methods for surveying gene expression, which rely on averaging the signal across a population of cells. Here, we employ single cell RNA sequencing in order to measure gene expression profiles at single-cell resolution. This allowed us to distinguish between estrogen-responsive and unresponsive populations and identify defined expression signatures for each. Also, because cellular responses are asynchronous, we were able to use the snapshot of expression profiles to infer the transcriptional changes as cells respond to estrogen and become increasingly disorganized. These techniques revealed not only the processes that may contribute to the earliest stages in the formation of estrogen-driven pre-cancerous cells, but also identified biomarkers of that transition. We have confirmed that the protein GREB1 appears in the pre-cancerous cells and is present in the majority of human ovarian cancers.

## Introduction

Ovarian cancer is the deadliest gynecological cancer with an overall 5-year survival rate of only 45%. However, if detected early, this survival rate increases to 92% [1]. These statistics demonstrate the importance of understanding the initiating events of ovarian cancer to better develop strategies for prevention and early detection. To date, there are many known risk factors for ovarian cancer but the molecular events leading to transformation remain unclear. Meta-analysis of 52 epidemiological studies investigating menopausal hormone use and ovarian cancer risk found that 55% of women who developed ovarian cancer had also used estrogen replacement therapy [2]. Using a mouse model of ovarian cancer, 17β-estradiol (E2) was shown to accelerate onset of ovarian cancer as E2 treated mice reached their end-point 30–40 days before microscopic tumours were observed in control mice [3]. Wild-type mice exposed to exogenous E2 over 60 days (modelling hormone replacement therapy) developed dysplastic lesions on the ovarian surface epithelium (OSE) and fallopian tube epithelium (FTE) [3,4]. On the ovary, these lesions are characterized by an increased incidence of both columnar epithelial cells and stratified hyperplastic cells. On the oviduct, there was significant increase in both stratified and hypertrophic FTE. Recent use of unbiased genomic and proteomics approaches demonstrate that both OSE and FTE are capable of giving rise to epithelial ovarian cancer [5,6]. Since our lab showed that prolonged E2 exposure impacts both cell types and genome-wide transcriptional regulation of estrogen receptor targets in mouse FTE was recently investigated [7], we present the transcription dynamics of estrogen-induced dysplasia in primary cultures of mouse OSE to further advance our understanding of how estrogen can accelerate transformation from either cells of origin.

The E2-induced OSE dysplasias are reproducible using primary cultures of OSE cells, where areas of columnar OSE and foci of multi-layered cells can both be observed after 15 days of exposure to E2. Immunohistochemical (IHC) staining of mouse ovaries and immunofluorescent (IF) staining of OSE cultures showed that these distinct phenotypes are associated with opposing gene expression patterns [4]. These co-existing morphologies highlight the heterogeneity of the responses to E2, confounding conventional methods used to measure transcript and protein expression changes. Here, we use single-cell RNA-Sequencing (scRNA-Seq) to tease apart this heterogeneity and to understand the phenotypic trajectory of OSE cells throughout the process of foci formation then extend our main finding to mouse FTE and human ovarian cancer tissue.

## Results

### Single-cell profiling of OSE cells

**Fig 1:**
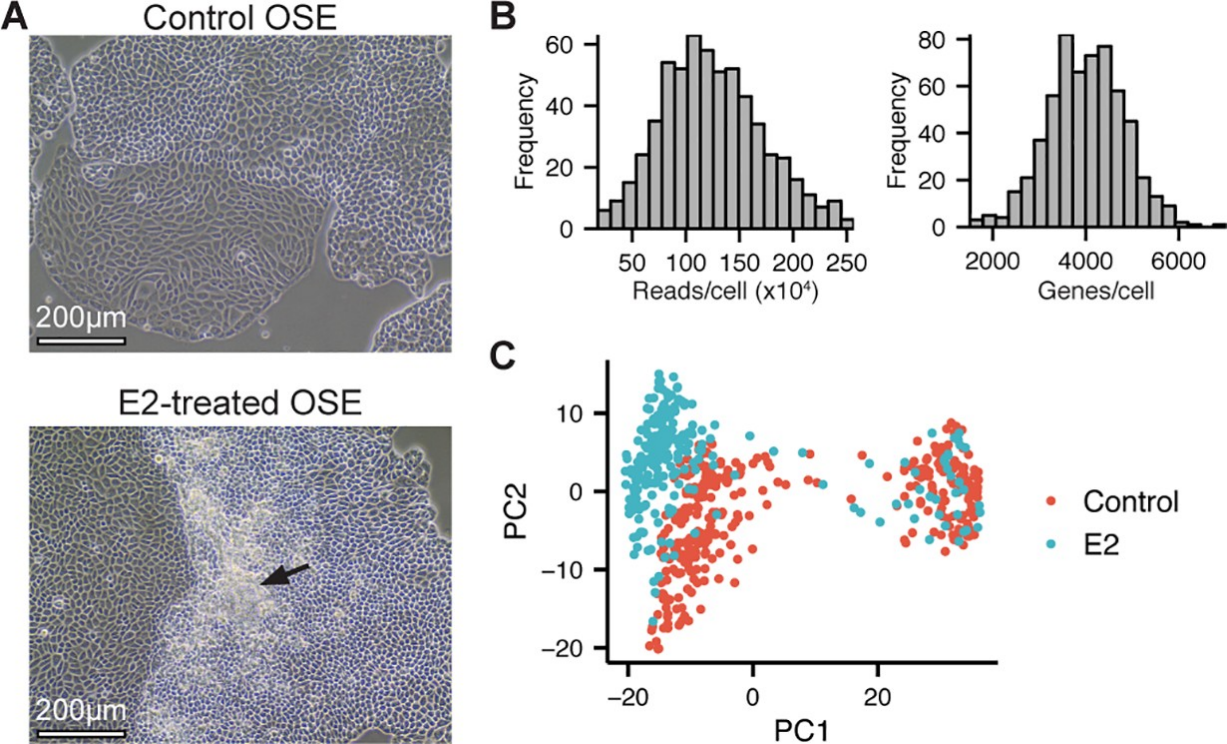
Single-cell profiling of control and dysplastic E2-treated OSE cells. (A) Phase-contrast images of control and E2-treated OSE cells. Scale-bar = 200μm. (B) Histogram of the number of reads (left) and genes (right) per cell following quality control filtering. (C) PCA of control and E2-treated cells from scRNA-Seq data. Data points represent individual cells and are coloured by treatment. 84.7% of total variance is accounted for by PC1 (75.6%) and PC2 (9.1%).

Substantial dysplasia was observed in primary cultures of OSE cells after prolonged E2 exposure, with disparate morphological changes present in the same dish (**Fig 1A**). To identify subpopulations of OSE and their responses to E2, we used scRNA-Seq to compare control cells and those that had been treated with E2 for 15 days. After processing and filtering out poor quality libraries (**Figure in S1 Fig**), the dataset comprised 329 control and 260 E2-treated cells, with a median of approximately 120,000 reads/cell and 4,000 genes/cell (**Fig 1B**).

Principal component analysis (PCA) revealed two distinct clusters of cells along the first principal component that did not segregate based on E2 treatment (**Fig 1C; Figure in S2 Fig**). PC2 and PC3, however, did correlate with the treatment. No known technical variable or typical biological source of variation (eg. cell cycle) correlated with these principal components, suggesting they represent biological variation of interest.

### Heterogeneity in OSE cell response to E2

**Fig 2:**
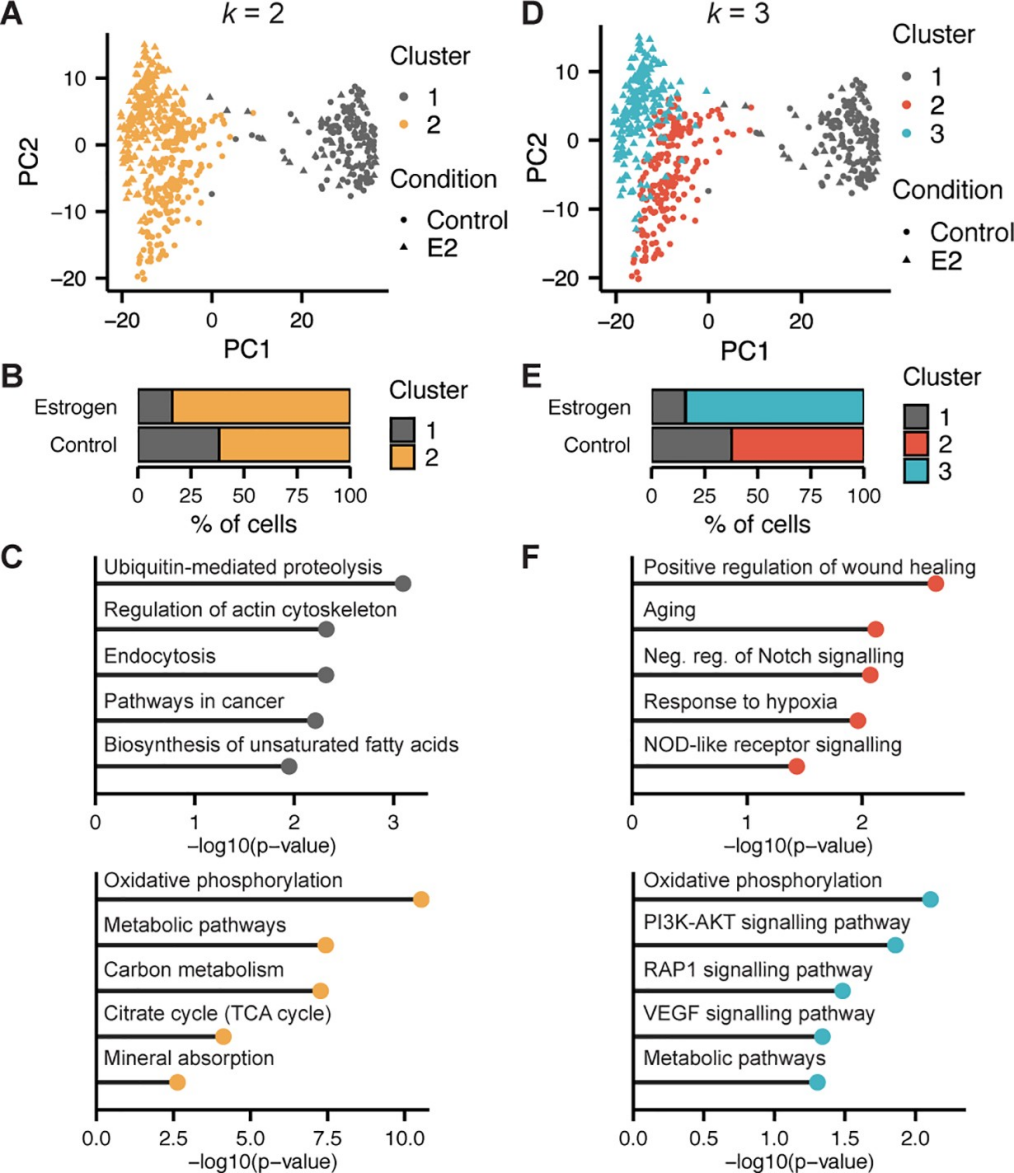
Unsupervised clustering reveals distinct heterogeneity in OSE. (A) PCA of OSE cells coloured by cluster (*k* = 2 clustering). (B) Stacked barplot displaying the percent of cells from each condition within each cluster. (C) Top KEGG Pathways and GO Terms (p-value < 0.05) associated with genes more-highly expressed in Cluster 1 (top) and Cluster 2 (bottom). (D) PCA of OSE cells coloured by cluster (*k* = 3 clustering). (E) Stacked barplot displaying the percent of cells from each condition within each cluster. (F) Top KEGG Pathways and GO Terms (p-value < 0.05) associated with genes more-highly expressed in Cluster 2 (top) and Cluster 3 (bottom).

To determine the gene expression differences that drive structure in the PCA, we used SC3 [8]—a consensus-based k-means clustering algorithm—to cluster the data. Given the two distinct populations along PC1, we first set *k* = 2 to define two clusters, splitting the mixed populations of cells along the first principal component (**Fig 2A and 2B**). We then used monocle [9] to identify 1,132 differentially expressed genes between clusters (**Table in S1 Table**).

Since the cells of the rightmost cluster did not spread along PC2 according to E2 exposure, while the cells of the left cluster did, we predicted that these two clusters capture differences in the cells’ ability to respond to E2. None of the additional principal components captured E2-associated differences in the cells of the right cluster and further sub-clustering does not separate the E2-treated cells from the untreated cells of this population **(Figure in S3 Fig),** suggesting these cells are effectively indistinguishable, despite differences in their culture conditions. The estrogen receptors *Esr1* and *Esr2* were detected in 23.4% and 4.9% of all cells, respectively, and *Gper1* (G protein-coupled estrogen receptor 1) was not detected in any cell (**Figure in S4 Fig**). *Esr1* is the predominant mediator of the transcriptional effects of E2 signalling in the OSE [10] and was expressed significantly higher in the leftmost cluster (*q* = 0.02). Together, this suggests that the right cluster represents a population of cells that are E2-unresponsive, despite having some degree of *Esr1* expression. To further support that the distribution of cells along the first principal component corresponds to estrogen responsiveness, we scored each cell based on their expression of genes comprising the “Early estrogen response” hallmark gene set from the Molecular Signatures Database [11]. All cells of the rightmost cluster, regardless of whether they had been exposed to E2, had lower gene set scores (*t*-test *p* = 9.16x10^-160^**; Figure in S4 Fig)**.

Functional enrichment analysis of differentially expressed genes between the right and left clusters was performed to explore the biological differences between these two clusters (**Table in S2 Table**). The E2-responsive cluster seems more metabolically active than the unresponsive cluster, as demonstrated by the top ontology terms for this cluster **(Fig 2C)**. The E2-unresponsive cluster was characterized by regulation of Actin cytoskeleton and, surprisingly, pathways in cancer **(Fig 2C)**. However, the majority of genes in this term are involved in evading apoptosis (*Xiap* [12], *Bmp4* [13]). Although some pro-proliferative signals were present (*Kras*, *Hras*, and *Araf)*, there was also significant up-regulation of *Pten*, a tumour suppressor that is sufficient to inhibit the proliferative signals of KRAS in OSE [14]. Cells in this cluster also have high expression of *Actin*, *Mmp2*, and *Rac1*—key genes for cell motility, filopodia, and lamellipodia formation. Thus, cells in the E2-unresponsive cluster are predicted to be mesenchymal in morphology and more quiescent than E2-responsive cells.

The initial decision to produce two clusters was based on the observed PCA structure; however, we found that *k* = 3 produced optimal clusters, maximizing the cells’ average silhouette width. Interestingly, the E2-unresponsive cluster was retained (Cluster 1), while the E2-responsive cluster was split into two clusters of almost exclusively control (Cluster 2) or E2-treated (Cluster 3) cells (**Fig 2D and 2E**). A total of 189 genes were differentially expressed between the two condition-specific clusters (Clusters 2 and 3) (**Table in S3 Table**). Control cells of the responsive cluster (Cluster 2) had higher expression of genes involved in wound healing, aging, response to hypoxia, negative regulation of *Notch* signalling, and NOD-like receptor signalling. E2-treated cells (Cluster 3) had an up-regulation in genes involved in metabolic, *PI3K-Akt*, *VEGF*, and *Rap1* signaling pathways (**Fig 2F**) (**Table in S4 Table**).

### Distinct cell populations *in vitro*

**Fig 3:**
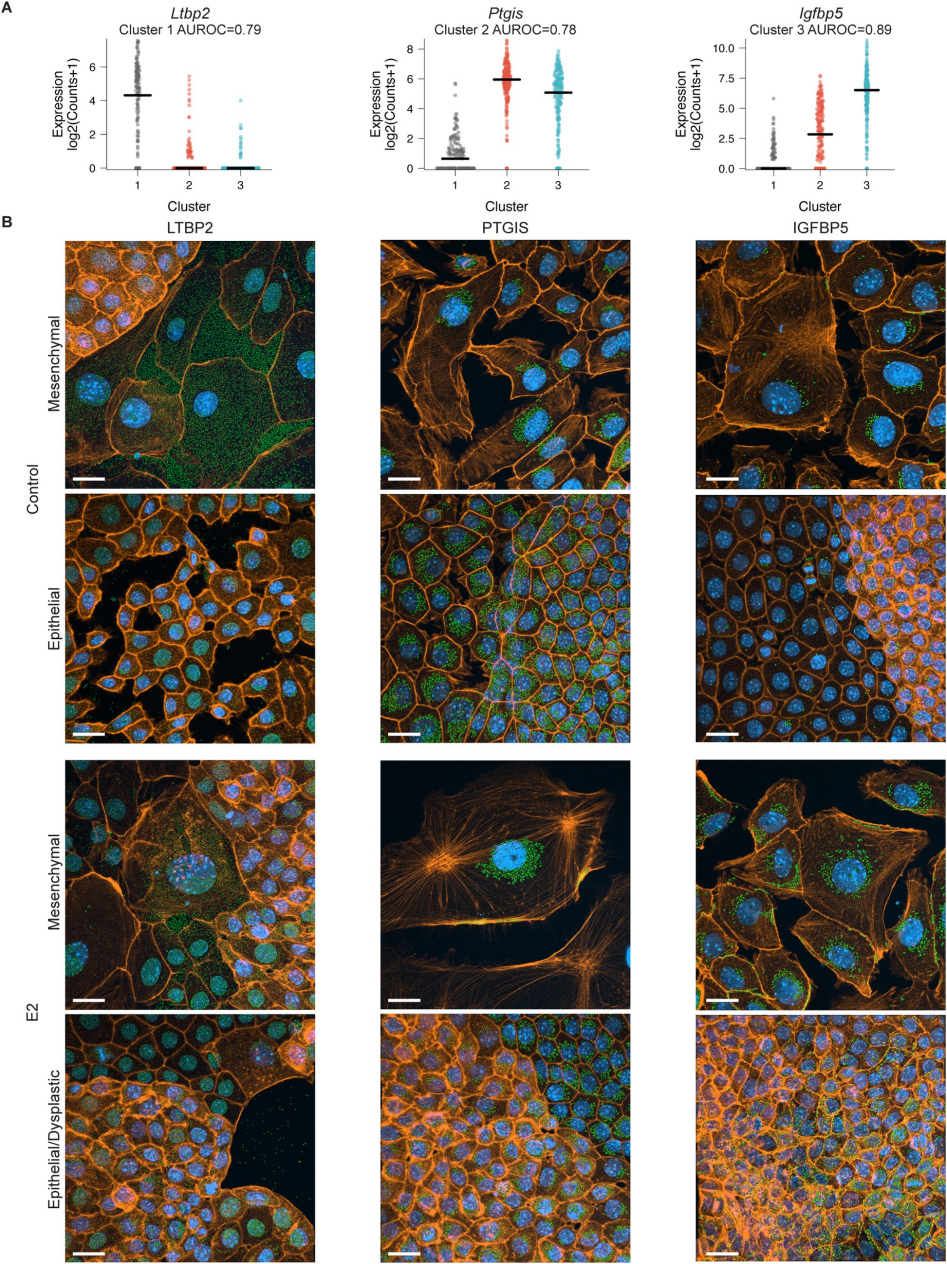
Cluster marker validation *in vitro* by IF. (A) Distribution of the logged counts of cluster markers *Ltbp2* (Cluster 1), *Ptgis* (Cluster 2), and *Igfbp5* (Cluster 3) for cells across each cluster. Horizontal bar represents the median expression value and AUROC values are given to show the strength of each gene as a marker for the corresponding cluster. (B) Control and E2-treated OSE cells stained for LTBP2 (n = 9–10), PTGIS (n = 13), and IGFBP5 (n = 9–10) by IF. Green = marker, orange = Actin, blue = DAPI. Scale-bar = 20μm.

To confirm that the clustering patterns identified in the scRNA-Seq data represented distinct populations of cells *in vitro*, we defined marker genes for each cluster (**Fig 3A; Table in S5 Table**). Protein expression encoded by the top marker genes *Ltbp2* (unresponsive cluster), *Ptgis* (control-specific cluster), and *Igfbp5* (E2-specific cluster) was assessed using IF **(Fig 3B; Figures in S5–S7 Figs**). LTBP2, a secreted protein, was detected in the cytoplasm and extracellular matrix surrounding control and E2-treated cells that had a flat, mesenchymal appearance (in contrast to the cobblestone, epithelial appearance of most OSE). PTGIS is an endoplasmic reticulum membrane protein that contributes to the “cellular response to hypoxia” ontology term in control cells (**Fig 2F**). It was modestly expressed in all cells, with a typically perinuclear localization. Expression was highest in control OSE cells where PTGIS was detectable throughout the cell cytoplasm. IGFBP5 was mostly absent in control OSE, but was predominant in E2-treated OSE. These results support the existence of 3 populations of cells in the *in vitro* model of E2-induced dysplasia, as predicted by PCA, where high expression of PTGIS and IGFBP5 are observed in the majority of cells in control and E2-treated cultures, respectively. This also supports that both control and E2-treated cultures have a subpopulation of mesenchymal OSE cells that uniquely expresses LTBP2. Consistent with this population being more mesenchymal, we found that the hallmark gene set “TGFB signalling” was dramatically higher in this cluster (*t*-test p = 1.17x10^-107^; **Figure in S8 Fig**).

**Fig 4:**
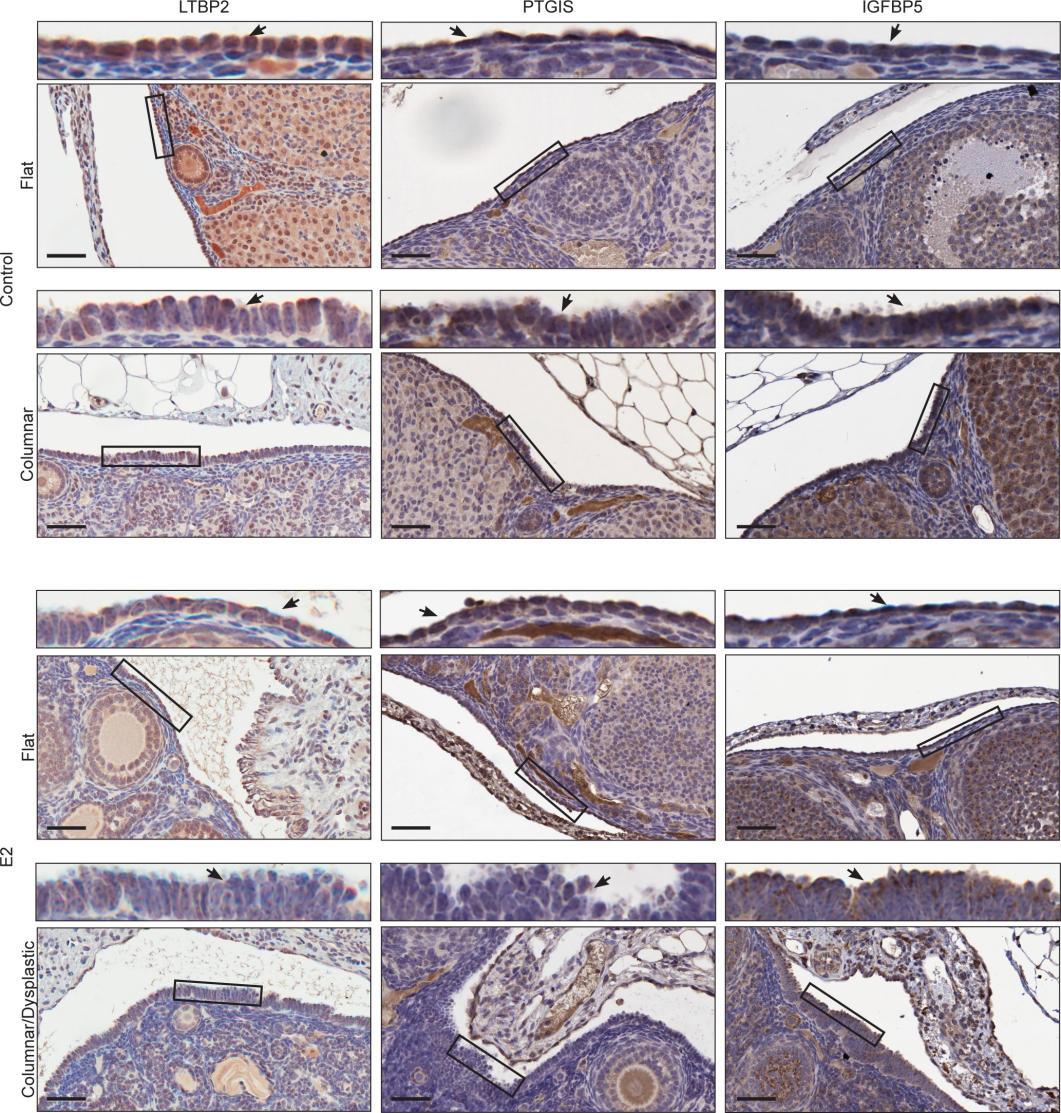
Cluster marker validation *in vivo* by IHC. IHC staining of ovaries from placebo and E2-treated mice for LTBP2 (placebo n = 3; E2 n = 5), PTGIS (placebo n = 5; E2 n = 5), and IGFBP5 (placebo n = 5; E2 n = 5). For each image, a higher magnification view of the OSE is provided above. Scale-bar = 50μm. See supplementary (**Figure in S9 Fig**) for IHC “no primary” controls.

To determine if these subpopulations are found *in vivo*, ovaries from mice treated with exogenous E2 were evaluated by IHC with these same markers **(Fig 4)**. E2-treated mice had significant areas of columnar and stratified OSE, as previously reported [3,4], and these cells had faint or absent LTBP2 and PTGIS expression, but strong punctate apical staining of IGFBP5. Placebo mice remain subject to endogenous estrus cycle activity and some areas of columnar OSE were observable, but the majority of OSE were squamous to cuboidal. PTGIS and LTBP2 were present at varying levels in the squamous to cuboidal cells, while IGFBP5 was consistently faint. These findings are largely consistent with the expression patterns predicted from the scRNA-Seq and IF data.

### Pseudotime analysis reveals phenotypic divergence

The formation of dysplastic regions is an asynchronous process: following E2 exposure, we observed both non-confluent monolayers and variably-sized foci of dysplastic cells. Therefore, we hypothesized that sampling cells for scRNA-Seq at a single time point would capture cells at various stages of foci formation, allowing us to use pseudotime analysis to reconstruct the trajectory of transcriptional changes that occur throughout the process. The continuous distribution of cells along the principal components, rather than discrete clustering, supports this hypothesis.

**Fig 5:**
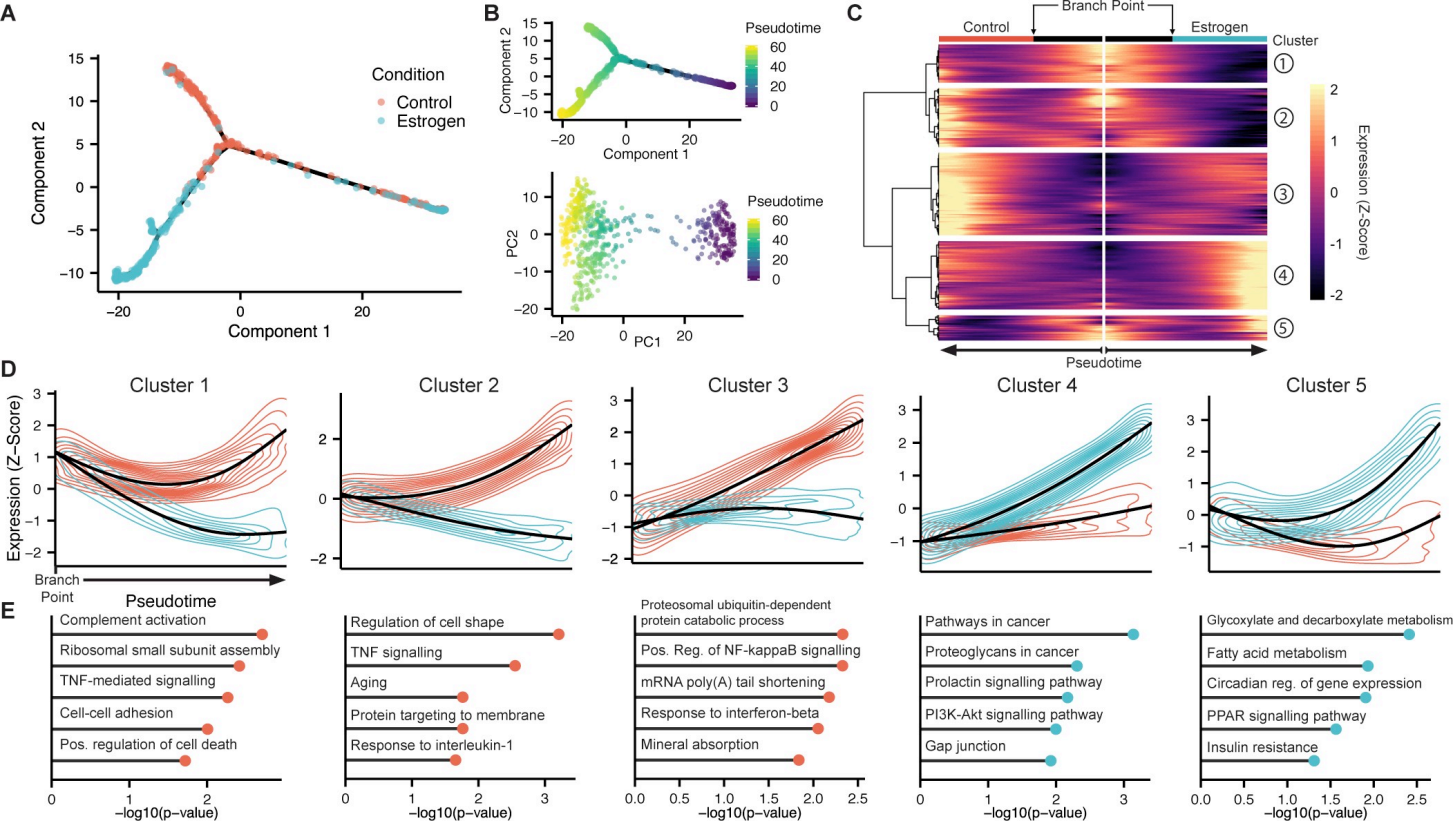
Trajectory analysis reveals phenotypic divergence of E2-treated cells. (A) Pseudotemporal trajectory of control and E2-treated OSE cells constructed by Monocle2. Data points are coloured by treatment. (B) The same trajectory (top) and PCA (bottom) coloured by the cells’ assigned pseudotime value. (C) Clustered heatmap of genes with branch-dependent gene expression as a function of pseudotime. The center of the heatmap represents the root cell (pseudotime = 0). Cells are ordered by increasing pseudotime value, diverging outwards from the heatmap’s center, corresponding to the two condition-specific trajectories. (D) Expression dynamics of genes from each cluster. The black line represents the average scaled (Z-score) expression values, which is surrounded by the two-dimensional kernel density estimate. Values are separated by branch (control-dominated branch: red; estrogen: blue). (E) Top KEGG Pathways and GO Terms (p-value < 0.05) associated with the genes of each cluster.

We used monocle [9] to construct a transcriptional trajectory of all the cells, naive to the condition that the cells came from. This trajectory was characterized by a branch common to cells from both conditions (representing the E2-unresponsive cluster) that diverges from a branch point into two condition-specific branches (**Fig 5A**). This can be interpreted as a linear trajectory for each condition, where depending on whether the cells had been exposed to E2, the trajectory diverges into a different state space. If the columnar and stratified states were separate “lineages” arising from a common precursor cell, we would expect a branched pattern in the trajectory of E2-treated cells, and if they arose from two distinct cell populations, we would expect two disconnected trajectories in E2-treated cells. Given that cells form a connected trajectory, and that each culture condition’s trajectory seems linear, this data supports the hypothesis that the columnar and stratified phenotypes represent different stages of OSE cell dysplasia along a common transcriptional path. Additionally, the trajectory connects the E2-unresponsive population with the responsive population, suggesting that cells can transition between these two states.

To characterize the divergent phenotypes in the trajectory, we used monocle [9] to pseudotemporally order the cells, ranking their distance along the trajectory from the cell of the E2-unresponsive branch farthest away from the branch point (**Fig 5B**). While pseudotime is often used to reconstruct temporal, albeit asynchronous processes, such as differentiation, we only use it here to define the relationship between cells along a phenotypic continuum. In this context, the defined root state is not meant to represent the starting point of a biological process, and unidirectionality is not assumed as cells transition through this continuum.

After assigning a pseudotime value to cells, we identified 693 genes with branch-dependent gene expression dynamics (**Fig 5C**; **Table in S6 Table**). Clustering of these genes based on their expression dynamics identified groups with common expression patterns (**Fig 5D**). We found that OSE cells cultured long-term in a steroid-free environment acquire more characteristics of cell stress as they diverge from the branch point. There is induction of genes associated with cell aging, inflammatory pathways, positive regulation of cell death, and other immunogenic signals for apoptosis and clearance (**Fig 5E; Table in S7 Table**). E2-treated cells show either no change or a decrease in these genes, but show increased expression of genes involved in metabolic activity, pathways in cancer, and proliferation via PI3K-Akt signaling (**Fig 5E**). Additionally, E2-treated cells show decreased expression of genes associated with regulating cell shape and cell-cell adhesion, which likely contributes to the development of dysplasia.

### The pseudotemporal trajectory models foci formation

**Fig 6:**
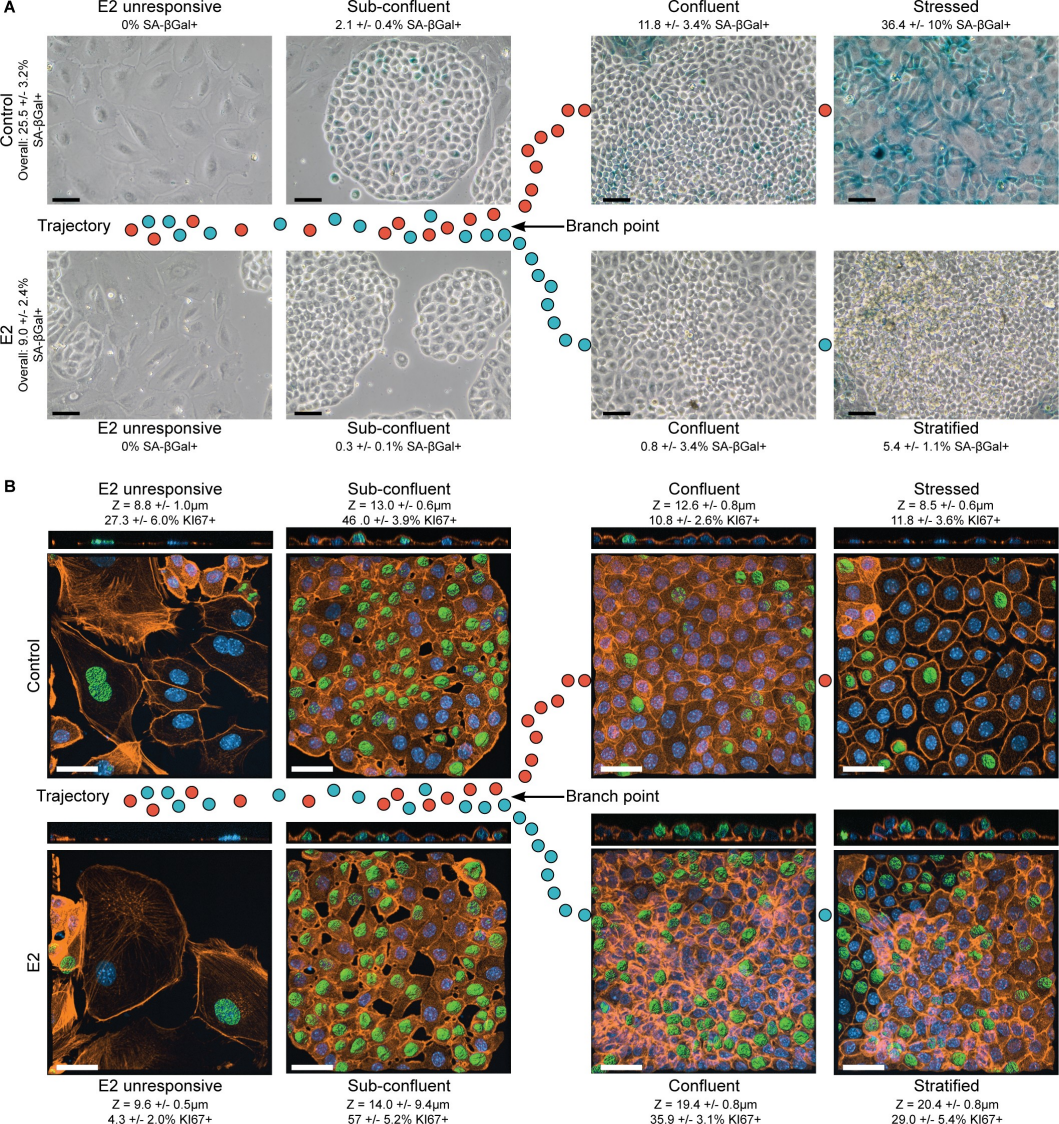
E2 promotes survival and proliferation. Control and E2-treated OSE cells stained for SA-βgal (A) and KI67 by IF (B). (A) Overall %SA-βgal, n = 7 using 4x objective; %SA-βgal/morphology, n = 6–7 using 40x objective. Scale-bar = 50μm. Representative images shown is a result of phase-contrast overlaid with bright-field. (B) Green = KI67, orange = Actin, blue = DAPI. %KI67+ = KI67+nuclei/DAPI; n = 6. Z = cell height; n = 5–6. Scale-bar = 30μm. Representative images display z-stack maximal intensity projections of Actin and DAPI channels merged with KI67 surface rendering. Axial view of field is also shown. (A-B) mean+/-S.E.M.

While the characteristics of the transcriptional trajectory are consistent with the observed biology of the culture system, we sought to validate that the structure of the trajectory relates to foci formation in the E2-treated cells and cell stress in the control cells. To do this, senescence-associated β-galactosidase (SA-βGal) activity was used as a marker for stressed cells [15–17], which we have previously shown identifies stressed OSE cells in culture [4]. Phase contrast images of the same field of view were overlaid to track cell morphology (**Fig 6A**). KI67 staining was used as a marker for proliferating cells [18], and actin staining was used to track cell morphology and approximate cell height (**Fig 6B**). E2-unresponsive cells occupying the common branch were SA-βGal-negative and sometimes KI67-positive in both control and E2-treated cultures. Also common between control and E2-treated cultures were areas of sub-confluent OSE where cells in small epithelial rafts or cells on the periphery of larger rafts were cuboidal in morphology, SA-βGal-negative, and ~50% KI67-positive. These areas of proliferative and cuboidal OSE cells, seen in both control and E2-treated cultures, are likely cells that exist shortly before phenotypic divergence. As the rafts become larger and more confluent, the divergence predicted by trajectory analysis becomes apparent. Confluent control cells remain similar in height to sub-confluent cells and remain SA-βGal-negative but become less KI67-positive. The final phenotype observed in control cultures, and presumably the last stage in the control-dominant branch of the trajectory, was SA-βGal-positive cells that remain mostly KI67-negative and have become enlarged and flattened in morphology, characteristic of senescent cells [19]. In contrast, E2-treated cells remain proliferative at confluence and either progressively assume a columnar morphology (as demonstrated with increased cell height) or become stratified. Consistent with the few E2-treated OSE cells existing in the control branch of the trajectory, some areas of SA-βGal-positive cells are seen in E2-treated cultures, but SA-βGal-negative columnar and stratified cells are the predominant phenotype.

**Fig 7:**
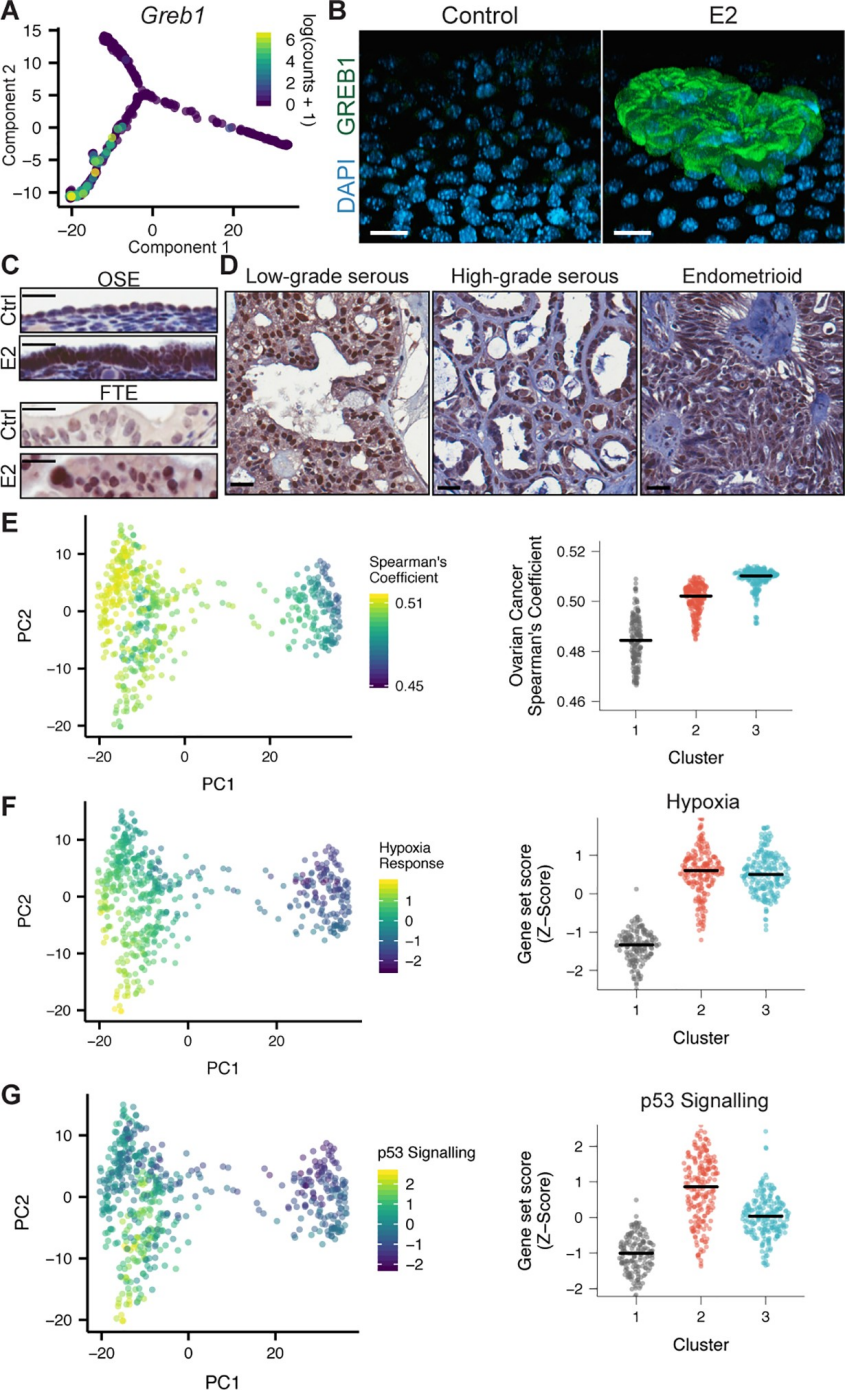
Prolonged E2 exposure leads to up-regulation of GREB1 in stratified OSE cells. (A) Trajectory of OSE cells, coloured by the logged *Greb1* counts. (B) Control and E2-treated OSE cells stained for GREB1 by IF. Scale-bar = 15μm. Representative images display z-stack maximal intensity projections of GREB1 and DAPI channels rotated 45° along Y-axis to best display image in XYZ dimension. n = 6–8. (C) IHC staining of GREB1 in the OSE and FTE from placebo and E2-treated mice (OSE: n = 4; FTE: n = 3) and three subtypes of human ovarian cancer (D): low-grade serous (n = 4), high-grade serous (n = 16), and endometrioid (n = 20). Scale-bar = 25μm. (E) Left: PCA plot of scRNA-seq data with each cell coloured by its Spearman’s correlation coefficient with human ovarian cancer cells from a high-grade serous ovarian tumour [22]. Right: The distribution of correlation values across the three clusters. Horizontal bars represent the median value for each cluster. (F,G) Left: PCA plot of scRNA-seq data with each cell coloured by “Hypoxia” (F) and “p53 Signalling” (G) gene set scores. Right: The distribution of gene set scores across the three clusters. Horizontal bars represent the median value for each cluster.

When exploring the expression patterns of all genes from the “Early estrogen signalling” gene set, we noted that, of all genes, *Greb1* had the most strikingly exclusive expression within the branch putatively associated E2-induced foci. *GREB1* is a known E2-responsive gene that we have previously shown to be over-expressed in human ovarian cancer relative to healthy human OSE and is known to promote cancer progression [20,21]. In the trajectory, *Greb1* was only present in E2-treated cells beyond the branch point and was detected in only 37% of E2-treated cells (**Fig 7A**). IF staining of OSE cultures demonstrate that all control cells are GREB1-negative and GREB1 is only present in E2-induced stratified OSE **(Fig 7B**). Together, these data suggest that the modelled trajectory represents the process of E2-induced foci formation where E2-treated cells first become columnar then progress into stratified OSE that are uniquely GREB1-positive. To further validate that the trajectory is representative of foci formation *in vivo*, we stained ovaries and oviducts of E2-treated mice and found increased expression of GREB1 in both stratified OSE and FTE (**Fig 7C**). If, in humans, similar dysplastic populations give rise to ovarian tumours, a similar gene expression pattern may be expected. We stained 40 human ovarian tumour samples and found detectable GREB1 in 75–85% of each of the various histological subtypes (**Fig 7D**).

Assessing a more general similarity to human disease, we also constructed a representative expression profile of human ovarian cancer cells by averaging the expression values from a recent scRNA-seq study of a human high-grade serous ovarian tumour [22] and correlated this profile with each cell in our study. This revealed that as cells form foci, their expression profile becomes more similar to human ovarian cancer cells (**Fig 7E**). Also, while E2-treated foci and confluent control cells both display a hypoxia response expression signature (**Fig 7F**), E2-treated cells fail to activate p53 signalling (**Fig 7G**). This may contribute to their sustained proliferation and could also drive genetic instability. Along with the activation of cancer-associated pathways as cells progress through this trajectory, these data support that the molecular profile of dysplastic regions may be consistent with pre-cancerous lesions in humans.

## Discussion

**Fig 8:**
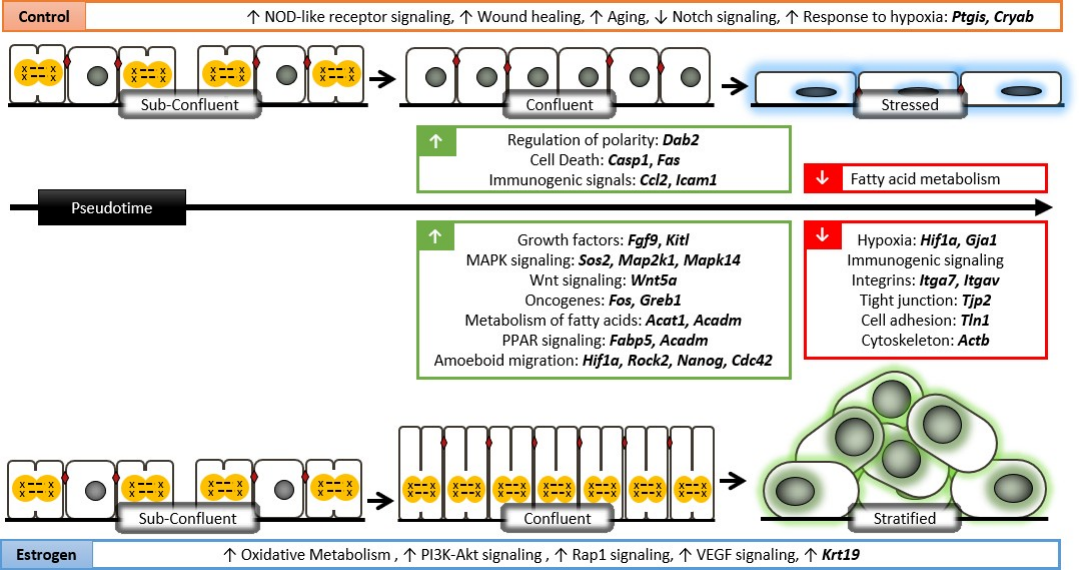
Molecular signaling mechanisms of estrogen-induced dysplasia in OSE. Sub-confluent cells from control and E2 cultures are phenotypically similar where they are proliferative and metabolically active. With increasing confluence, cell-cell adhesions form between control cells and proliferation is slowed due to contact inhibition mediated by regulators of polarity. Without E2, control cells become stressed with increasing confluence and upregulate genes associated with hypoxia, cell death, and inflammatory responses involving immune cell recruitment through the TNF signalling pathway. E2 treated cells suppress inflammatory immunogenic signals, overcome hypoxia via up-regulation of *Hif1a*, show up-regulation of growth factors, mediators of MAPK/Wnt/PI3K-Akt signaling, known oncogenes, and metabolism. With persistent proliferation, columnar cells are first observed to maximally accommodate proliferative cells in an already confluent monolayer. While *Krt19* is consistently higher in E2 treated OSE cells, these cells show loss of epithelial architecture and upregulate key genes involved in amoeboid migration to detach from the ECM and move through dense hypoxic conditions.

The OSE monolayer is well-documented as a heterogeneous population with multiple morphologies observable in both *in vitro* primary cultures and *in vivo* models [23]. Full appreciation of the heterogeneous nature of OSE cells was made possible using scRNA-Seq and, in doing so, we have resolved the asynchronous response to estrogen that OSE cells display, resulting in the formation of dysplastic lesions **(Fig 8)**.

In the *in vitro* model of E2-induced OSE dysplasia, where control and E2-treated OSE cells are expanded in culture over 15 days, we propose that sub-confluent cells from both cultures are phenotypically similar where they are proliferative, metabolically active, and cuboidal. With increasing confluence, cell-cell adhesions form between control cells and proliferation is slowed due to contact inhibition mediated by regulators of polarity, such as *Dab2* [4]. Without E2, control cells become stressed with increasing confluence and upregulate genes associated with hypoxia (*Ptgis*, *Cryab* [24]), cell death (*Casp1*, *Fas* [25]), and inflammatory responses involving immune cell recruitment through the TNF signalling pathway (*Ccl2*, *Icam1*) [26,27]. E2-treated cells appear to overcome hypoxia via up-regulation of E2-responsive *Hif1a* [28] and *Connexin 43* (*Gja1*) [29,30]. Inflammatory immunogenic signals were suppressed in E2-treated cells, suggesting that E2 may contribute to an immune-restricted microenvironment, allowing pre-neoplastic lesions to develop and evade immune surveillance [31]. In addition to bypassing stress and apoptosis, E2-treated cells show up-regulation of growth factors (*Fgf9*, *Kitl*), mediators of MAPK signaling (*Map2k1*, *Mapk14*), Wnt signaling (*Wnt5a*), PI3K-Akt signaling, and *Fos* (a known E2-responsive oncogenic driver of proliferation and differentiation [32,33]). This increased proliferation is likely sustained by increased metabolism, where E2-treated OSE activate PPAR signaling to promote fatty acid oxidation and mitochondrial enzymes (*Acat1*, *Acadm*) that can utilize these fatty acids in the Krebs cycle [12].

*Krt19* was consistently higher in E2-treated OSE cells in all analyses performed. While this suggests that E2 promotes epithelial differentiation, components necessary for proper monolayer formation such as integrin (*Itga7*, *Itgav*), tight junction (*Tjp2)*, cell adhesion (*Tln1*), cytoskeleton (*Actb*) genes were all decreased in E2-treated cells. By confocal microscopy, E2-induced columnar cells observed in confluent areas consistently outnumber confluent control cells in a given field of view, suggesting that increased cell height maximally accommodates proliferative cells in an already confluent monolayer while still allowing attachment to the basement membrane. With increased proliferation associated with progressive loss of epithelial architecture, E2-treated cells also upregulate key genes involved in amoeboid migration (*Rock2* [34], *Nanog* [35], *Cdc42* [36]). This phenotype can be driven by *Hif1a* [37] and has been shown to be the preferred mode of migration of epithelial cancer cells to detach from the ECM and move through dense hypoxic conditions, providing a possible explanation for OSE stratification.

Given the lack of strategies for early detection of ovarian cancer and consequent poor patient prognosis, investigating genes upregulated throughout E2-induced dysplasia formation may inform development of diagnostic and prognostic markers for E2-driven and -responsive tumours. *Igfbp5* [38–40], *Enpp2* [41], *Ctsh* [42,43], *Lpl* [44,45], and *Tacc1* [46–48] are the top five genes correlated with the E2 branch in pseudotime and all are currently under investigation as cancer biomarkers. Preliminary studies in various human pathologies have demonstrated that IGFBP5 [49], ENPP2 [50], ENPP2 metabolites [44] and LPL [44] are secreted factors detectable in serum. Given that they are up-regulated in pre-neoplastic cells, these are strong candidates to be investigated as markers for the early detection of ovarian cancer. *Greb1* stands out because it is a known E2-responsive gene in breast and ovarian cancer that drives tumour progression [20], and it is currently being investigated as an alternative prognosis marker for tamoxifen treatment [51]. *Greb1* has previously been shown to be E2-inducible in normal mouse FTE [7] and our present finding is the first to show E2 induction of GREB1 in OSE, where it is uniquely expressed in dysplastic cells after prolonged E2 exposure. Given that GREB1 expression is highest in dysplastic OSE and FTE, this suggests that GREB1 may play a role in the initiation of tumour formation in both cell types.

In this study, scRNA-Seq was used to provide a global mechanistic explanation for how prolonged E2-exposure can lead to OSE dysplasia and increased susceptibility to transformation, and to reveal potential biomarkers for early detection. Currently, the data remains largely correlative and additional biological assays are required to validate the model and delineate correlation from causation. Nevertheless, this study is the first in the field of ovarian cancer to explore the initiating events in the transformation of ovarian epithelial cells at single-cell resolution. It provides a solid foundation for supporting current hypotheses and a framework for developing new strategies for ovarian cancer prevention and early detection.

## Materials and methods

### Ethics statement

Experiments involving mice were performed according to the Canadian Council on Animal Care Guidelines for the Care and Use of Animals on a protocol approved by the University of Ottawa Animal Care Committee. Mice in this study were euthanized using CO2. Tissue microarray of human ovarian cancers was obtained from the Cooperative Human Tissue Network (University of Virginia, U.S.A.).

### Mice and E2 pellet implant

Exogenous E2 was delivered to FVB/N mice and their tissue was collected as previously described (n = 5/treatment) [4].

### Primary isolations of mouse OSE, maintenance in culture, and E2 treatment

OSE cells were isolated from mouse ovaries, maintained, and treated with E2 as previously described [4,52]. OSE were maintained at 37°C in OSE media that consists of α-MEM media (Corning) with 5% fetal bovine serum, 2ng/mL epidermal growth factor (Sigma), and 0.01mg/mL insulin-transferrin-sodium selenite supplement (Roche). For E2 treatment of OSE, cells were seeded and allowed to normalize to hormone-free media consisting of 5% charcoal-stripped fetal bovine serum in phenol red-free DMEM-F12 media (Sigma) for 48 hours before treating with 100nM E2 (Sigma). An equivalent volume of 100% EtOH (vehicle) was added to control dishes for a final concentration of 0.0002% EtOH. Media was refreshed every 3–4 days and collected for single cell RNA-sequencing or immunofluorescent staining 15 days after E2 treatment. All control and E2-treated OSE cell experiments analyzed in this study are after 15 days in culture.

### Single-cell cDNA library preparation and sequencing

Cells were processed and sequencing libraries were prepared according to the Fluidigm C1 Single-Cell mRNA Seq HT IFC v2 protocol, using a medium (10–17μm) integrated fluidic circuit (IFC). For quality control, samples were stained with a LIVE/DEAD viability stain (ThermoFisher) and each capture site was visually inspected using an EVOS FL Cell Imaging System (ThermoFisher) to score the number and viability of cells in each capture site. Successful library tagmentation was assessed using the Advanced Analytical Fragment Analyzer and sample molarity was calculated using the average fragment size. Samples were pooled at equal molarity, spiked with 20% PhiX, and sequenced together in a single high-output 150 cycle NextSeq500 run with read 1 set to 26bp and read 2 set to 75bp.

### scRNA-Seq data processing

Fastq files corresponding to the 20 cDNA libraries were demultiplexed into 800 fastq files, each representing one of the 800 capture sites on the Fluidigm C1 IFC, using Fluidigm’s API. Transcripts were quantified using kallisto [53], and gene-level read counts were imported into R using the tximport package [54]. The scater package [55] was used for quality control, normalization, and exploratory analysis of the data.

### scRNA-Seq data filtering

Only capture sites annotated to contain one live cell were kept. Cells with less than 20,000, or more than 250,000 reads were removed to eliminate poor quality libraries, and capture sites that may contain multiple cells, respectively. Cells with a high or low proportion of detected genes (>3 median absolute deviations—MADs—from mean) and those with a high proportion of mitochondrial reads (>3 MADs) were removed. Lastly, only genes that were detected in a minimum of 10 cells were retained. The final expression matrix included the expression of 14299 genes across 636 cells.

### Normalization and imputation of expression values

The R package scran [56] was used to calculate scale factors for each cell as described previously [57]. Each cell’s expression profile was then scaled by this factor. To restore structure in the data that may be lost as the result of drop-out characteristic of scRNA-Seq, we performed data imputation using the MAGIC algorithm [58] with the following parameters: n_pca_components = 20, t = 4, ka = 3, k = 9, epsilon = 1. These values were used for downstream clustering and pseudotime analysis.

### Clustering and differential expression between clusters

Imputed expression values were used to define cell clusters. k-means-based consensus clustering was performed using SC3 [8] to segregate the cells into 2 or 3 clusters. The optimal number of clusters (k = 3) was determined by finding the value of k that maximized average silhouette width. k = 2 segregated the two large groupings of cells along the first principal component, so we also explored this clustering pattern in downstream analysis. Differential expression analysis was performed using monocle [9] to fit a generalized linear model to the expression values of each gene and filtering for genes with a q-value < 0.05 and a log2 fold change > 0.5.

### Functional enrichment analysis

The DAVID Gene Functional Classification Tool [59,60] was used to explore KEGG pathways and GO terms significantly associated with differentially expressed genes between clusters in PCA and clusters of genes with similar expression dynamics throughout pseudotime. The top 5 terms ranked by EASE p-value were determined by subjecting each cluster to identical filtering parameters. Terms were removed if there was intracluster term conflict, (eg. both positive and negative regulation of apoptosis appearing in the same cluster), if term is not unique to one cluster, if the biological process had 10 or more child terms (to eliminate broad and uninterpretable processes), or if they could be reasonably agreed upon as not applicable to the model system (eg. liver regeneration, Alzheimer's disease, etc.). KEGG pathways were given priority because they provide the most information for biological interpretation of data. Full list of unfiltered GO Terms are made available in **Tables in S2, S4, and S7 Table**.

### Identification of marker genes of each cluster

Marker genes for each cluster were identified using SC3 [8], which constructs a binary classifier for each gene based on the mean expression of the gene in each cluster. The area under the receiver operator characteristic curve (AUC) is used to determine the accuracy of the gene as a marker for the cluster. The AUC values were ranked, and top-ranking genes for each cluster were chosen for validation by IF based on commercial availability of antibody and quality of stain (assessed by preliminary antibody optimization experiments).

### Pseudotime analysis of ovarian epithelial cell populations

A pseudotime trajectory of all cells was constructed using the DDRTree method implemented in monocle2 [61]. The root branch was defined as the branch comprising cells from each condition. A generalized linear model was fit to the expression values with an interaction term to model branch-specific expression dynamics as a function of pseudotime. Because the imputation and normalization eliminate a large amount of variation, the interaction coefficients for each gene were used to identify differentially expressed genes (>2 standard deviations from mean of all coefficients).

### Hallmark Gene Set Scoring

Hallmark gene sets were acquired from the Molecular Signatures Database [11]. Each cell was scored based on its expression of the genes within each gene set using the AddModuleScore function in the R package Seurat [62]. For each cell, this function determines the average relative expression of each gene of the gene set compared to groups of expression level-matched control genes. These relative expression values were then standardized using a Z-score transformation.

### Correlation with ovarian tumour scRNA-seq data

Processed scRNA-seq data was retrieved from Winterhoff *et al*. [22] Expression values were log-transformed and scaled before using the highly variable genes from the manuscript to cluster the cells into two groups using hierarchical clustering. Similar to the data presented in the original publication, this separated the population into a CD24+ cluster of cancer cells, and a CD24- cluster of stroma cells. The expression profiles of all cancer cells were averaged to produce a representative expression profile of human ovarian cancer cells. Using genes present in both the mouse and human data sets, this expression profile was correlated (Spearman’s rank correlation) with each cell of our data set.

### Data and code availability

All data are available at GSE121957 and analysis notebooks are hosted at https://github.com/dpcook/scRNASeq-Estrogen.

### Immunofluorescence (IF) staining

Cells were seeded onto glass coverslips and treated with E2 for 15 days. Cells were fixed, permeabilized, blocked, and probed according to antibody datasheet instructions, then mounted onto slides using ProLong Gold mountant with DAPI (ThermoFisher Scientific). See Supp.Table.8 for details on antibodies used for IF. All IF experiments have at least two independent experimental replicates and “n” refers to fields of view.

### Immunofluorescence microscopy and analysis

Confocal images were acquired using a Zeiss LSM 800 confocal microscope system using Plan-Apochromat 40X/1.3NA oil objective. Images were processed using the Imaris Image Analysis Software v8 (Bitplane). IF images are shown as z-stack maximum intensity projections of DAPI (blue) or Actin (orange) channels. Protein of interest (green) is represented as solid surface objects created based on the dynamic range of all control and E2 treated images acquired within the biological replicate. The surface object intensity threshold was set based on the maximal intensity value of the dynamic range. Control slides processed without primary antibody and original unmerged images are included as supplementary (**Figures in S5–S7 Figs**). %KI67+ cells was determined using ratio of KI67+nuclei/total nuclei (DAPI) (n = 6). Cell membrane co-staining was not ideal due to permeabilization required for IF antibodies so Actin was used as an alternative means to estimate cell height (n = 6). Cell height was determined by Actin surface rendering in Imaris. Due to mounting of coverslip, cell height was generally limited to <~20μm.

### Senescence-associated (SA)-βgal Stain

Cells were seeded onto glass coverslips and treated with E2 for 15 days. Cells were fixed with 4% paraformaldehyde (w/v, in PBS) for 10 mins, then incubated with SA-βGal staining solution [63] overnight at 37°C. Coverslips were mounted onto slides using ProLong Diamond antifade mountant (ThermoFisher Scientific) and allowed to cure for 24 hours. Slides were visualized using an EVOS 507 XL Core imaging system under phase-contrast and brightfield to best capture morphology and SA-βGal stain, respectively. Quantification of SA-βgal was performed using Fiji version 1.0. SA-βgal-positive (SA-βgal+) cell pixels were quantified using automated colour deconvolution (Methyl Green DAB vector, colour 1) on brightfield images. SA-βgal- pixels were quantified by subtracting bright-field pixels from the corresponding phase-contrast pixels using Image Calculator. %SA-βgal+ cells was determined using ratio of SA-βgal+ cell pixels/total cell pixels.

### Immunohistochemical (IHC) staining of tissue

IHC was performed using a previously described protocol [3]. IHC staining was performed on ovaries from 3–5 mice per treatment group and on a tissue microarray of human ovarian cancers obtained from the Cooperative Human Tissue Network (University of Virginia, U.S.A.). See **Table in S8 Table** for details on antibodies used for IHC. No-primary control sections are included in **Figure in S9 Fig**. Images were acquired using ScanScope CS2 (Leica Biosystems, Concord, Canada).

## Supporting information

S1 Fig scRNA-Seq quality control and imputation. (A) Boxplots of each library’s transcript counts relative to number of cells observed in corresponding capture sites. (B) Scatter plot of *Col1a2* (mesenchymal gene) and *Krt19* (epithelial gene) expression levels (left) and PCA of OSE cells (right) before imputation and (C) following imputation using MAGIC.
(TIF)

S2 Fig Principal component analysis of control and E2-treated OSE. Scree plot (left) for the principal component analysis highlighting the fraction of total variance explained by the first few principal components, along with PCA plots of PC1 vs. PC3 (middle) and PC4 (right).
(TIF)

S3 Fig Clustering patterns with increasing *k*. PCA of OSE cells, coloured by cluster. Increasing the number of clusters (*k* parameter) does not separate the control and E2-treated cells of the putative E2-unresponsive population.
(TIF)

S4 Fig Assessing E2 responsiveness. Top row: PCA of OSE cells, coloured by the logged counts of *Esr1* (left), *Esr2* (middle), and a gene set score for the “Early Estrogen Response” gene set from the Molecular Signatures Database (right). Bottom row: Plot showing the distribution of expression levels (logged counts) within cluster. Horizontal bar represents the median expression value for each cluster.
(TIF)

S5 Fig Staining control with no primary antibody and original images for LTBP2 IF. Original merged and unmerged z-stack maximum intensity projections from the DAPI, AF555 (Actin), and AF488 (LTBP2) channels for LTBP2 staining. Scale bar = 15μm.
(TIF)

S6 Fig Staining control with no primary antibody and original images for PTGIS IF. Original merged and unmerged z-stack maximum intensity projections from the DAPI, AF555 (Actin), and AF488 (PTGIS) channels for PTGIS staining. Scale bar = 15μm.
(TIF)

S7 Fig Staining control with no primary antibody and original images for IGFBP5 IF. Original merged and unmerged z-stack maximum intensity projections from the DAPI, AF555 (Actin), and AF488 (IGFBP5) channels for IGFBP5 staining. Scale bar = 15μm.
(TIF)

S8 Fig TGFB1 signalling in OSE. Left: PCA of OSE cells coloured by a gene set score of “TGFB1 Signalling” from the Molecular Signatures Database. Right: The distribution of gene set scores between the three clusters. Horizontal bar represents the median value for each group.
(TIF)

S9 Fig IHC staining controls. Tissue sections prepared with no primary antibodies for LTBP2, IGFBP5, PTGIS, and GREB1 in the ovary and fallopian tube epithelial (FTE).
(TIF)

S1 Table Differential expression results between Clusters 1 (rightmost cells) and 2 (leftmost cells; k = 2). (XLS)

S2 Table Full list of GO Terms and KEGG Pathways associated with Clusters 1(rightmost cells) and 2 (leftmost cells; k = 2). (XLS)

S3 Table Differential expression results between Clusters 2 and 3 (k = 3). (XLS)

S4 Table Full list of GO Terms and KEGG Pathways associated with Clusters 2 and 3 (k = 3). (XLS)

S5 Table Area under receiver operator characteristic (ROC) curves. (XLS)

S6 Table Pseudotime branch-dependent gene expression results. (XLS)

S7 Table Full list of GO Terms and KEGG Pathways associated with each cluster of branch-dependent genes. (XLS)

S8 Table List and details for antibodies used. (XLS)
