# Supplementary figures and images for: Single-cell RNA-sequencing reveals transcriptional dynamics of estrogen-induced dysplasia in the ovarian surface epithelium
Source: PLoS Genet. 2018 Nov 12;14(11):e1007788. doi: 10.1371/journal.pgen.1007788 (PMC6258431; doi:10.1371/journal.pgen.1007788)

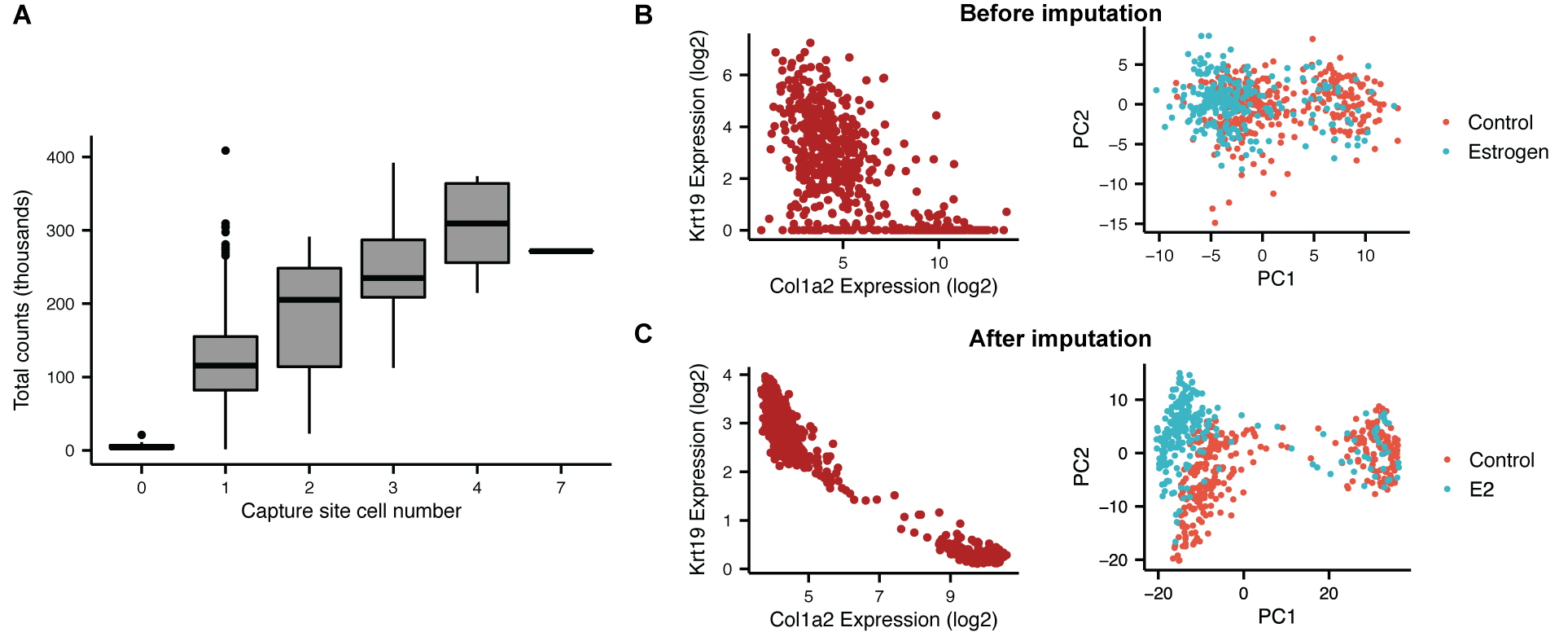

Supplement: S1 Fig — (A) Boxplots of each library’s transcript counts relative to number of cells observed in corresponding capture sites. (B) Scatter plot of Col1a2 (mesenchymal gene) and Krt19 (epithelial gene) expression levels (left) and PCA of OSE cells (right) before imputation and (C) following imputation using MAGIC. (TIF) [file pgen.1007788.s001.tif]

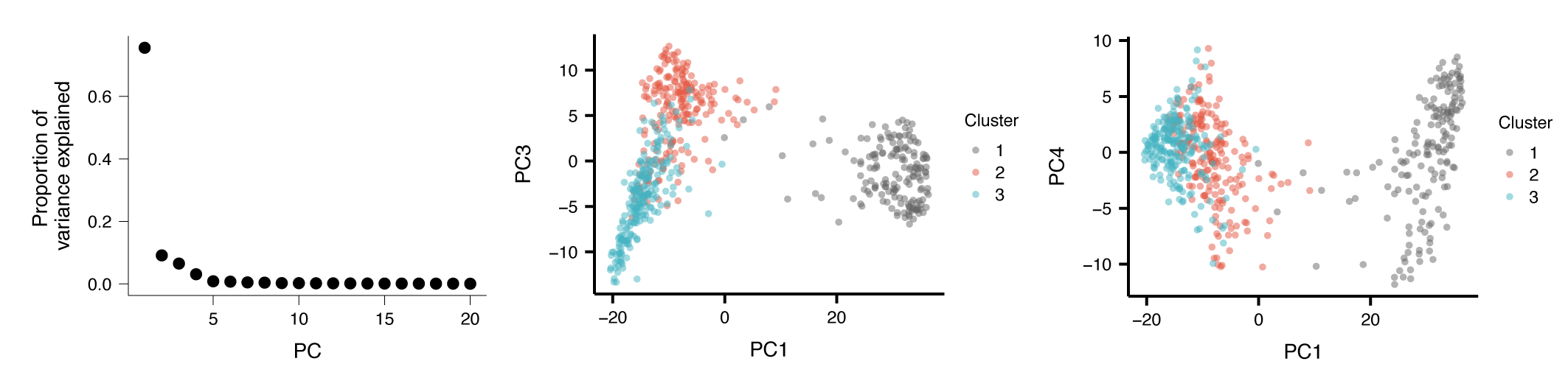

Supplement: S2 Fig — Scree plot (left) for the principal component analysis highlighting the fraction of total variance explained by the first few principal components, along with PCA plots of PC1 vs. PC3 (middle) and PC4 (right). (TIF) [file pgen.1007788.s002.tif]

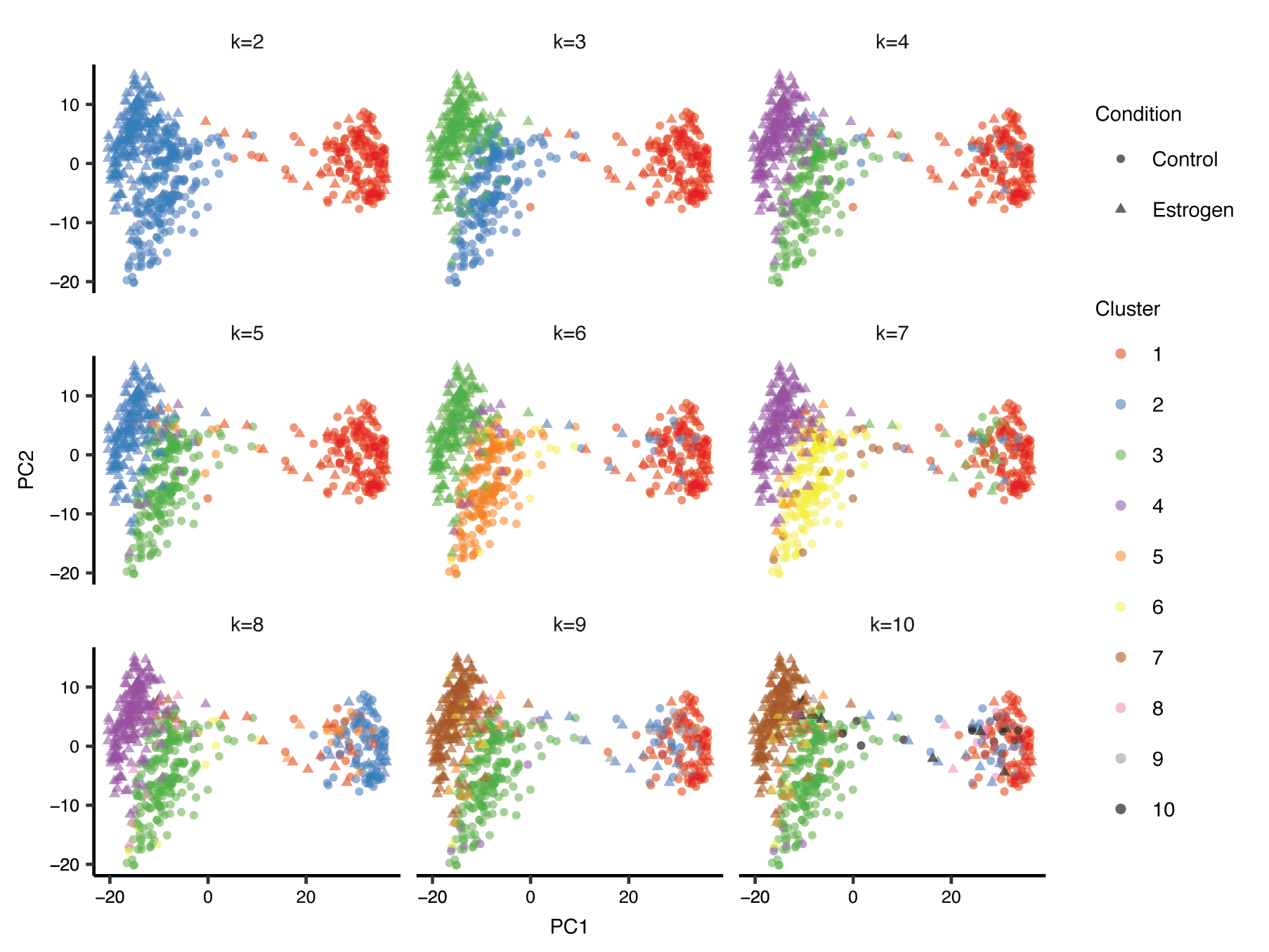

Supplement: S3 Fig — PCA of OSE cells, coloured by cluster. Increasing the number of clusters (k parameter) does not separate the control and E2-treated cells of the putative E2-unresponsive population. (TIF) [file pgen.1007788.s003.tif]

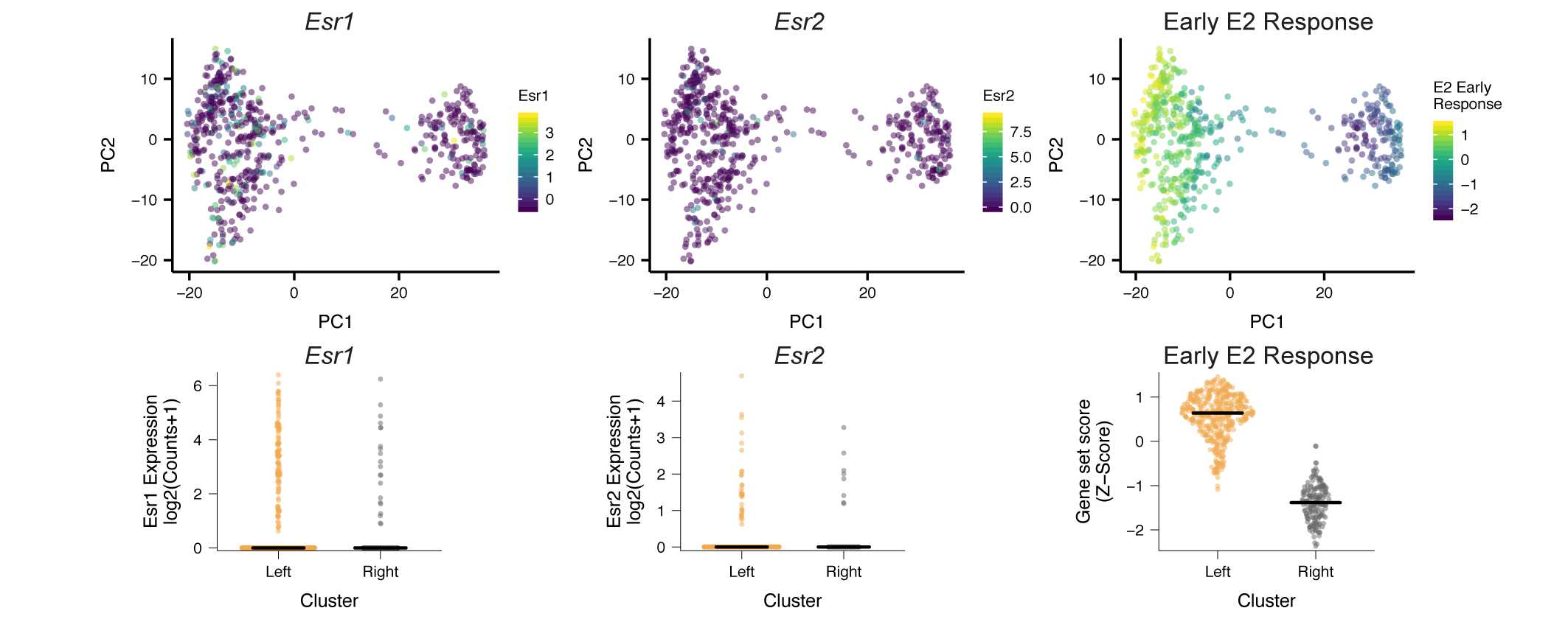

Supplement: S4 Fig — Top row: PCA of OSE cells, coloured by the logged counts of Esr1 (left), Esr2 (middle), and a gene set score for the “Early Estrogen Response” gene set from the Molecular Signatures Database (right). Bottom row: Plot showing the distribution of expression levels (logged counts) within cluster. Horizontal bar represents the median expression value for each cluster. (TIF) [file pgen.1007788.s004.tif]

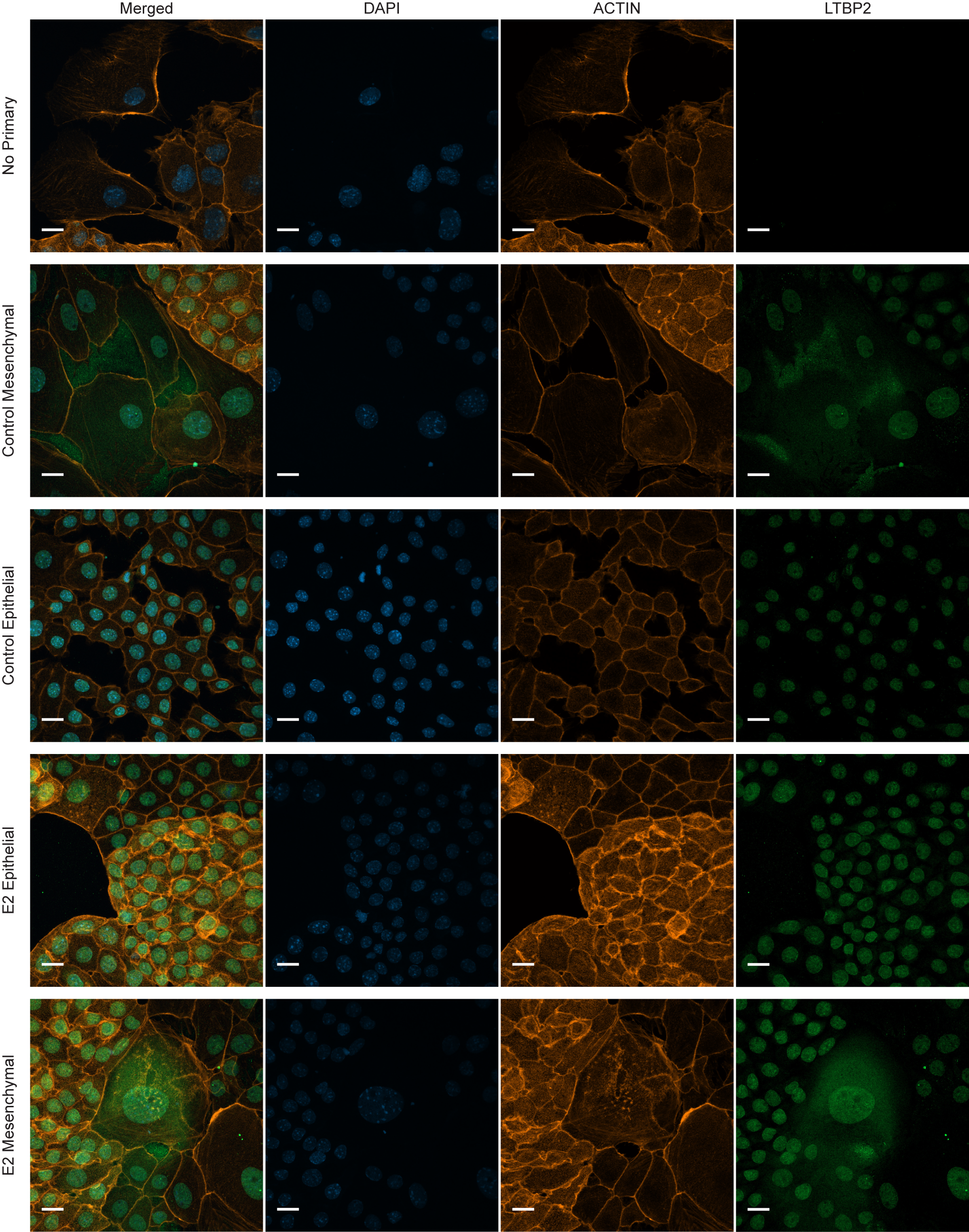

Supplement: S5 Fig — Original merged and unmerged z-stack maximum intensity projections from the DAPI, AF555 (Actin), and AF488 (LTBP2) channels for LTBP2 staining. Scale bar = 15μm. (TIF) [file pgen.1007788.s005.tif]

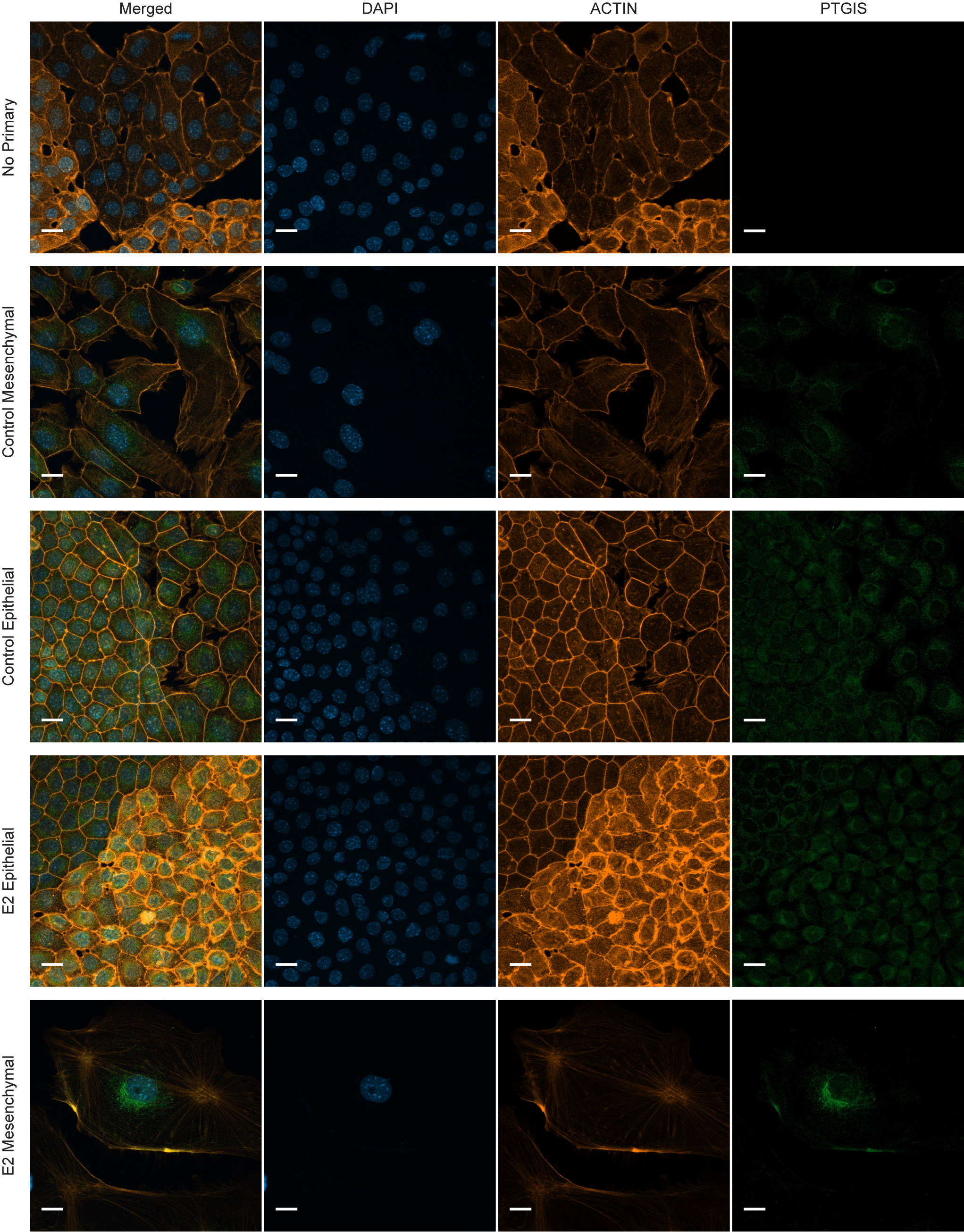

Supplement: S6 Fig — Original merged and unmerged z-stack maximum intensity projections from the DAPI, AF555 (Actin), and AF488 (PTGIS) channels for PTGIS staining. Scale bar = 15μm. (TIF) [file pgen.1007788.s006.tif]

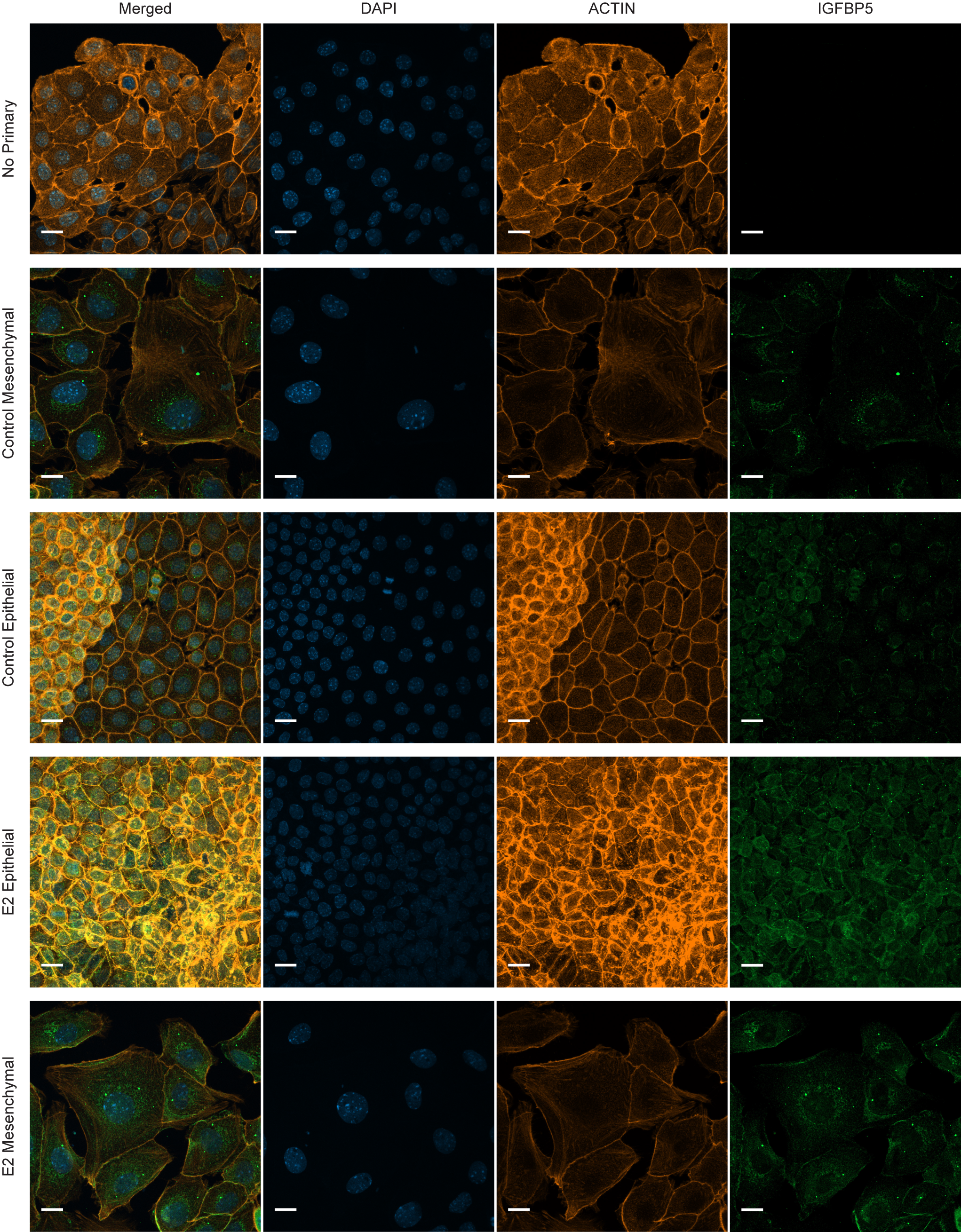

Supplement: S7 Fig — Original merged and unmerged z-stack maximum intensity projections from the DAPI, AF555 (Actin), and AF488 (IGFBP5) channels for IGFBP5 staining. Scale bar = 15μm. (TIF) [file pgen.1007788.s007.tif]

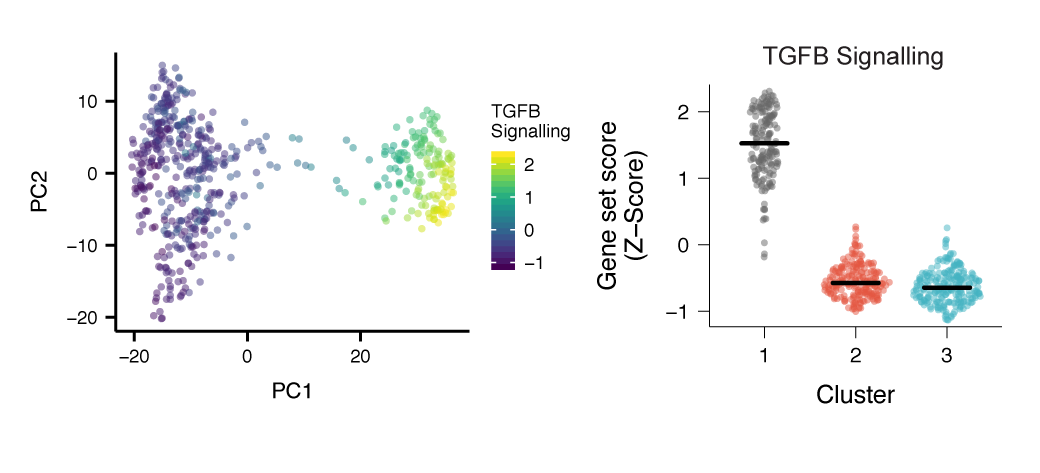

Supplement: S8 Fig — Left: PCA of OSE cells coloured by a gene set score of “TGFB1 Signalling” from the Molecular Signatures Database. Right: The distribution of gene set scores between the three clusters. Horizontal bar represents the median value for each group. (TIF) [file pgen.1007788.s008.tif]

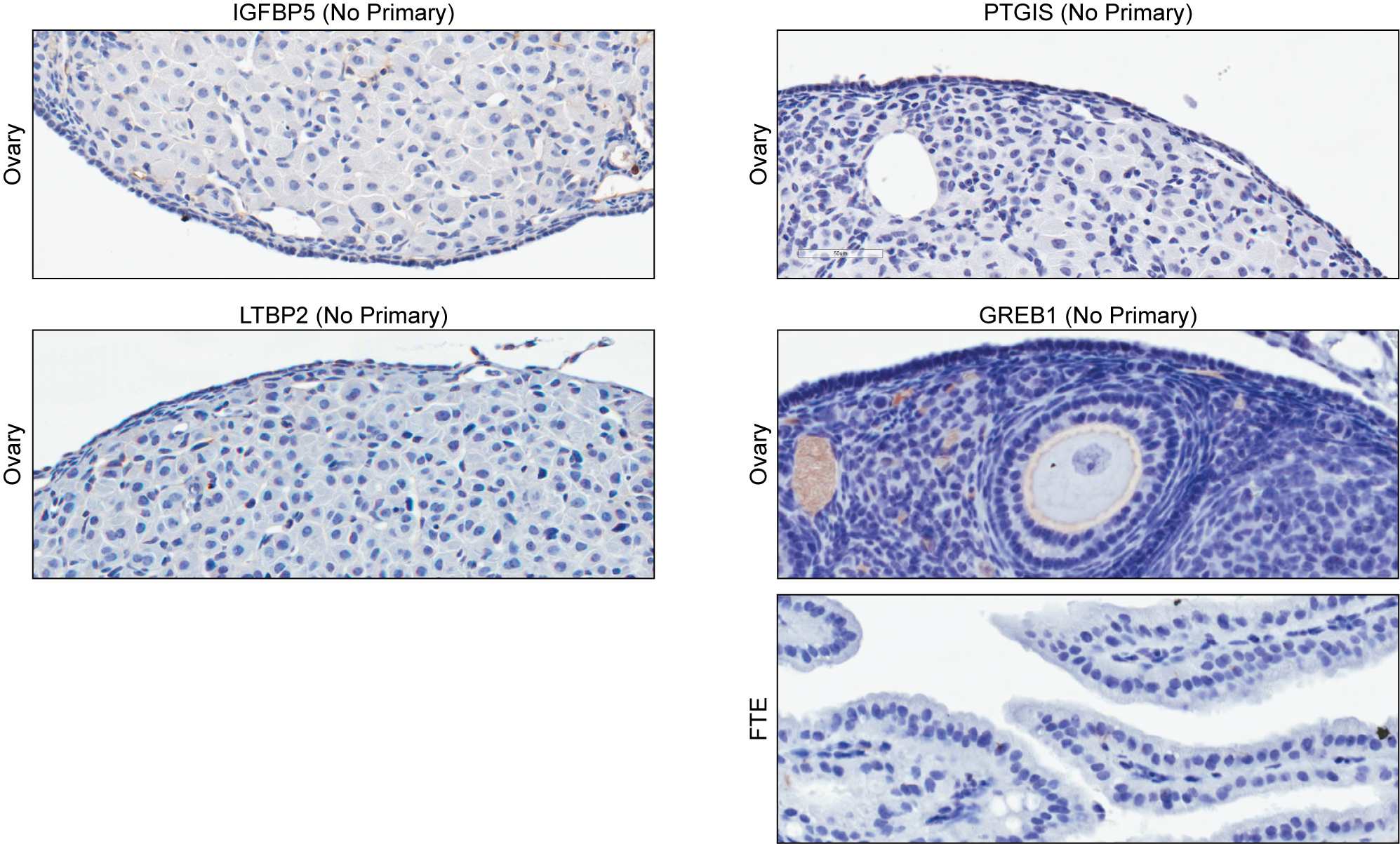

Supplement: S9 Fig — Tissue sections prepared with no primary antibodies for LTBP2, IGFBP5, PTGIS, and GREB1 in the ovary and fallopian tube epithelial (FTE). (TIF) [file pgen.1007788.s009.tif]
